# Metal-metal interactions in catalysis from spatial separation to physical mixtures

**DOI:** 10.1038/s41467-026-71870-6

**Published:** 2026-04-14

**Authors:** Rena Oh, Man He, Jian Yang, Liang Zhao, Shi-Gang Sun, Xiaoyang Jerry Huang

**Affiliations:** 1 Center of Advanced Electrochemical Energy, Institute of Advanced Interdisciplinary Studies, School of Chemistry and Chemical Engineering, School of Energy and Power Engineering, Chongqing University, Chongqing, China; 2 BASF Advanced Chemicals Co. Ltd, Shanghai, China

**Keywords:** Synthesis and processing, Chemical engineering, Heterogeneous catalysis

## Abstract

Physical mixtures of segregated metals recently demonstrated enhanced activity in redox reactions by assigning specific elementary steps to distinct sites to close the catalytic cycle through efficient transfer of reaction intermediates across conductive or reductive supports. Here, we delineate the fundamental shift from single static alloy sites to dual static separated structures, and ultimately to a dual dynamic mode. We term this mechanism dynamic interfacial cooperative catalysis. While collisions between identical single-sites provide no catalytic enhancement, collisions between distinct metal phases actively drive the reaction by facilitating cross-site electron and intermediate transfers. This dynamic synergy offers a blueprint for non-alloyed designs in high-concentration environments and energy-storage systems, including redox flow batteries.

## Abstract (web summary)

Separating metals can outperform mixed atomic sites by promoting different steps of a reaction. This perspective defines a dynamic cooperative mode in which collisions between distinct metal phases transfer electrons and intermediates to boost redox cycles.

## Introduction

Heterogeneous catalysis underpins modern chemical manufacturing^1,2^, enabling processes that span energy conversion^3,4^, environmental remediation^5,6^, and the synthesis of fuels and fine chemicals^7–10^. Achieving high catalytic performance has long relied on the rational integration of multiple active components, with efficient cooperation between distinct catalytic sites being a central objective in catalyst design^11–13^. In supported metal nanoparticle systems, such cooperation is particularly critical for facilitating complex reaction networks composed of coupled elementary steps, traditionally addressed through metal-metal interactions within alloyed catalysts^14–17^.

**Fig. 1:**
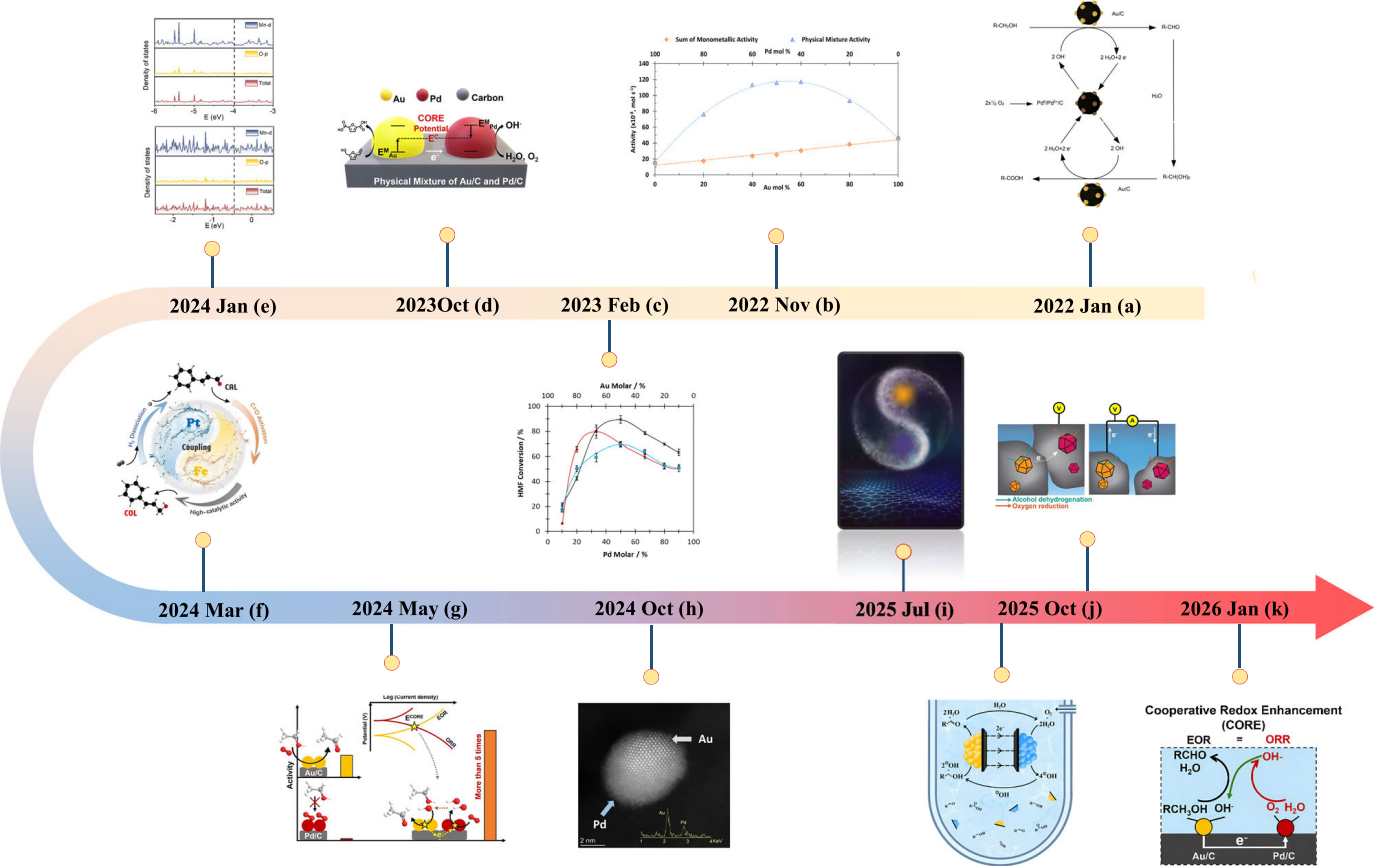
Publication trends in physical mixture catalysts from Huang and Hutchings’ group over the past four years. **a** Cooperative redox enhancement (CORE) of Au-Pd^18^; **b** CORE kinetics^35^; **c** Effect of Au-Pd Ratio on CORE^36^; **d** Polarized catalytic sites boost thermochemical rates^37^; **e** Physical mixtures between semiconductor and conductor^26^; **f** Synergy from Pt and Fe physical mixtures^23^; **g** Tafel analysis predicts CORE^38^; **h** Solvent-free environment^21^; **i** CORE review^9^; **j** Bimetallic catalysis driven by CORE^19^; Galvanic coupling predicts CORE^20^; **k** Designing bimetallic catalysts via CORE^39^. The representative figure of each publication and its published year are indicated in the figure.

Recently, however, attention shifted towards catalyst structures that deliberately disconnect the interactions between different metals, moving away from designs that force their atomic-scale proximity^9,18–20^. This shift led to the emergence of catalyst classes collectively described as spatially separated^21–24^ or physical mixture catalysts^18,25–30^, in which different metals are positioned at distinct locations within a shared catalytic environment. These designs challenge the long-standing assumption that intimate atomic-level integration is a prerequisite for effective metal-metal cooperation^31–34^. The publication trend over the past four years from Huang and Hutchings’ group is shown in Fig. 1^9,18–21,23,26,35–39^.

One major pathway for metal-metal separation is found in spatially separated catalysts that work through cooperative redox enhancement (CORE). In these systems, metal nanoparticles couple redox reactions while coexisting at a physical distance on a common support, often a carbon-based material^19,21,36,39^. A fundamental characteristic of these catalysts is that the reactions on different metals must occur at the same time, because they simultaneously catalyze the respective half-reactions of a single redox pair. The metal-metal separation can maintain the redox potential of each metal, enhance electron transfer between metal particles, and suppress site poisoning in thermo- and electrochemical 5-hydroxymethylfurfural (HMF) oxidation reactions^37^ and thermo- and electrochemical nitrate reduction reactions^22,40^.

Spatially orthogonal catalysts, pioneered by Adam F. Lee and Karen Wilson, represent another sophisticated subclass of spatial separation. While they share the fundamental similarity of isolating active sites to prevent unwanted atomic-level intereference or alloying, they differ significantly in their temporal operation. Spatially orthogonal catalysts are built on a hierarchically interconnected macroporous-mesoporous architecture affording spatial compartmentalization of chemical functionalities by locating each active site in different types of pores in a common support particle. In this structure, catalysis on different metals must occur in a specific, sequential order as reactants travel through the pore hierarchy^41,42^. This method was first demonstrated using silica nanoparticles with interconnected Pd-functionalized macropores and Pt-functionalized mesopores to sequentially oxidize cinnamyl alcohol to cinnamaldehyde over Pd nanoparticles, and then to cinnamic acid over Pt nanoparticles^41^. This concept was expanded to acid-base catalysts to sequentially catalyze acid-catalyzed esterification over sulfated zirconia in a macropore and then base-catalyzed transesterification over magnesium oxide in a mesopore^43^. A key merit of this structure is its ability to promote specific reaction sequences in cascade catalysis while avoiding competing side reactions. However, spatial orthogonalization possesses inherent drawbacks, primarily the synthetic complexity required to engineer precise hierarchical porous supports and the strict structural constraints it imposes on molecular diffusion.

More recently, however, a fundamentally different concept emerged in the form of physical mixture catalysts. Building upon the simultaneous synergy observed in spatially separated systems, the physical mixture catalysts that we designed benefit from both CORE and dynamic interfacial cooperative catalysis (DICC). Counterintuitively, these systems, which have been long regarded as suboptimal due to the absence of fixed atomic-scale interfaces, demonstrated markedly enhanced catalytic performance across a wide range of thermo- and electrochemical redox reactions^9,18^. Unlike spatially separated catalysts or rigidly co-supported catalysts, physical mixtures do not rely on rigid hierarchical architectures or on sharing support particles between active sites. Instead, their activity originates from dynamic interactions between neighboring metal particles.

**Fig. 2:**
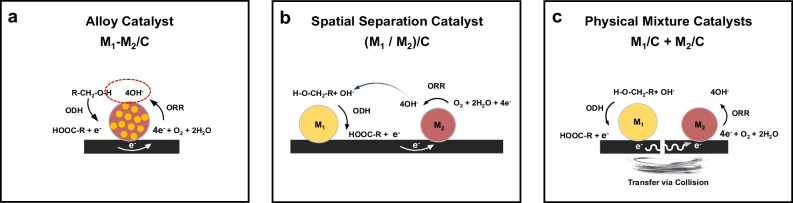
Structural evolution of metal-metal cooperation from static to dynamic catalytic frameworks in redox catalysis. **a** Metal-alloyed catalyst, **b** spatially separated, and **c** physically mixed catalytic systems.

In physical mixture catalysts, each metal functions as a selective active site for specific redox reactions that can be coupled through collisions, while efficient catalytic turnover is sustained through rapid transfer of reaction intermediates or charge across distinct conductive or reductive support particles^18^. Crucially, transient collisions and short-lived proximity between distinct metal particles are essential to the observed enhancement, transforming catalytic sites from “static entities” into “dynamically cooperative systems”. Recent theoretical frameworks, such as the CORE effect, successfully established the thermodynamic basis for electronic communication between metals via conductive supports, highlighting molecular spillover and electron transfer^18,19^ without the electronic compromise of alloys^44^. However, retaining this synergy stable for a long time requires a new conceptual model. We propose that these systems operate via DICC. Unlike the static connectivity in alloys or co-supported structures, DICC relies on the stochastic collisions between catalyst particles induced by multiple mechanisms such as mechanical stirring, magnetic stirring, ultrasound, and facilitates molecular spillover and charge transfer^45–47^. This distinction is not merely semantic but fundamentally a different concept: it shifts the design parameter from controlling atomic distance or spatial distribution as static variables of multifunctional catalyst to controlling collision frequency as dynamic variables between different monofunctional catalyst particles. The fundamental concept, evolving from alloy catalysts to spatially separated catalysts and ultimately to physically mixed catalysts, is illustrated in Fig. 2.

Spatially separated catalysts consist of metal nanoparticles deposited on a common support, where active sites remain spatially fixed but may undergo migration or restructuring via Ostwald ripening^48–50^. In contrast, physical mixture catalysts operate through collision-enabled, dynamically evolving interfacial interactions, in which active sites have a high probability of transient contact within the reaction medium, while retaining a low propensity for alloy formation^9^. This dynamic behavior allows physical mixture catalysts to adapt to diverse reaction environments, balance competing half-reactions, and sustain high activity even under high reactant concentrations.

In this perspective, we delineate the conceptual boundaries between spatial separation and physical mixtures as two divergent pathways for metal-metal mutual cooperation in heterogeneous catalysis. We first trace the evolution of cooperative catalyst design from alloying to spatial separation and ultimately to physical mixtures. We then examine the mechanistic origins of dynamic interfacial cooperation, highlighting how collision-enabled interactions fundamentally differ from static dual-site structures. Finally, we discuss key challenges and future opportunities, including the correlation of the nature and frequency of active-site interactions with catalytic performance, and a redefinition of the catalytic active site using the concept of physical mixture catalysts. We propose DICC as a distinct and generalizable concept to inspire new strategies for the rational design of cooperative monofunctional catalysts.

## Catalytic disassembly strategy for high-performance catalysis

**Fig. 3:**
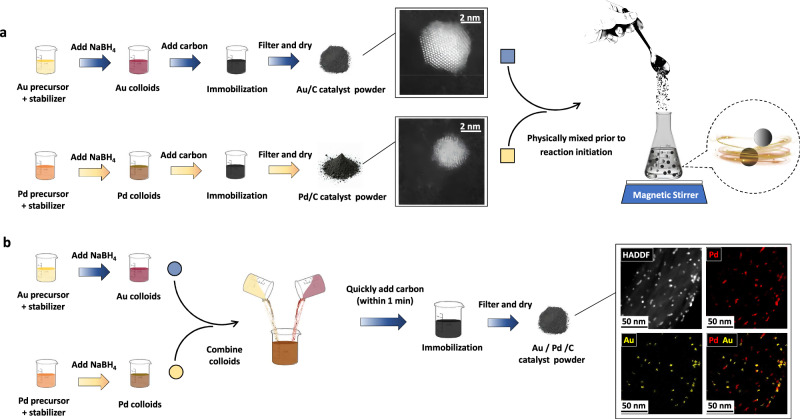
Synthesis protocols for metal-metal separated catalyst architectures. Preparation of (**a**) physical mixture catalyst and (**b**) spatially separated catalyst using sol-immobilization method, illustrated using carbon-supported Au and Pd nanoparticles as model components. High angle annular dark field-scanning transmission electron microscopy (HAADF-STEM) image of Au/C and Pd/C nanoparticle composing physical mixture catalyst are shown in (**a**). HAADF-STEM image with energy dispersive X-ray spectroscopy (XEDS) mapping of Au and Pd, marked by yellow and red, respectively, of Au/Pd spatial separation catalyst is shown together to confirm the metal-metal separation.

Alloy catalysts consist of two metallic elements combined at the atomic level. Particularly when the alloy catalyst involves Janus particles, core-shell structures, or specific morphologies, they require preparation methods such as co-precipitation, wet impregnation, solvothermal synthesis, or high-temperature reduction with precise control over metal ratios, temperature, and atmosphere^51–53^. Contrary to alloy catalysts, the catalytic disassembly strategy is comparatively simpler. This approach involves preparing catalysts with physical mixtures or spatially separated active components, avoiding the need for strict atomic-level alloying. As shown in Fig. 3a, carbon-supported Au and Pd nanoparticles are taken as a representative example to illustrate this strategy. The Au/C and Pd/C catalysts were prepared individually and mixed immediately prior to their application in the reaction. This approach, with reduced synthetic complexity and cost, holds strong potential for scale-up in industrial catalysis. Furthermore, the preparation of spatially separated catalysts is similar to the preparation of physical mixture catalysts: the metal colloids are first combined and then rapidly immobilized within 1 min on a common carbon support, yielding the catalyst denoted as (Au/Pd)/C (Fig. 3b).

The prepared catalysts, including physical mixtures can be applied in various reaction systems mainly in gas-phase and liquid-phase reactions. A key requirement for the preparation of physical mixture catalysts is to ensure intimate contact or near-homogeneous mixing of the individual components. In thermochemical systems, mixing can be achieved through several approaches, including magnetic stirring, mechanical agitation (e.g., using a robotic arm), and ultrasonic treatment. In fixed-bed reactors, quartz wool is commonly used to compress the catalyst bed, thereby maintaining close contact between the catalyst components. Furthermore, in electrochemical systems binders are often employed to immobilize powder catalysts on the electrode surface while simultaneously enhancing the proximity among active sites^54–56^. In this Perspective, we discuss the six-electron tandem oxidation of biomass-derived HMF to 2,5-furandicarboxylic acid (FDCA) as a model reaction to evaluate the performance of physical mixture and spatially separated catalysts. The full reaction pathway and intermediates 5-hydroxymethyl-2-furancarboxylic acid (HMFCA) and formyl-2-furancarboxylic acid (FFCA) are illustrated below^18^.

Step 1 $${{\rm{HMF}}}({{{\rm{C}}}}_{6}{{{\rm{H}}}}_{6}{{{\rm{O}}}}_{3})+{2{{\rm{OH}}}}^{-}\to {{\rm{HMFCA}}}({{{\rm{C}}}}_{6}{{{\rm{H}}}}_{6}{{{\rm{O}}}}_{4})+{2{{\rm{e}}}}^{-}+{{{\rm{H}}}}_{2}{{\rm{O}}}$$

Step 2 $${{\rm{HMFCA}}}+{2{{\rm{OH}}}}^{-}\to {{\rm{FFCA}}}({{{\rm{C}}}}_{6}{{{\rm{H}}}}_{4}{{{\rm{O}}}}_{4})+{2{{\rm{e}}}}^{-}+2{{{\rm{H}}}}_{2}{{\rm{O}}}$$

Step 3 $${{\rm{FFCA}}}+{2{{\rm{OH}}}}^{-}\to {{\rm{FDCA}}}({{{\rm{C}}}}_{6}{{{\rm{H}}}}_{4}{{{\rm{O}}}}_{5})+{2{{\rm{e}}}}^{-}+{{{\rm{H}}}}_{2}{{\rm{O}}}$$

**Fig. 4:**
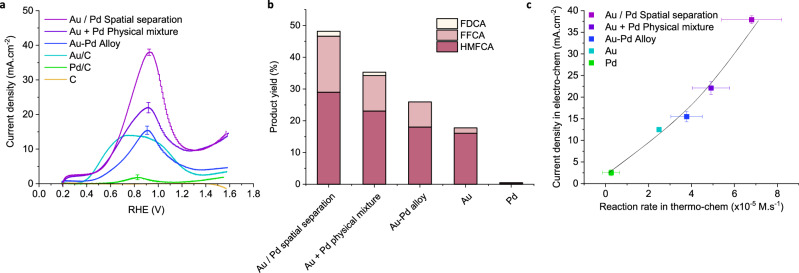
Performance comparison of divergent catalyst structures for electro- and thermochemical 5-hydroxymethylfurfural oxidation over Au and Pd catalysts. **a** Electrochemical cyclic voltammetry of HMF oxidative dehydrogenation over a series of Au and Pd catalysts, showing that the spatially separated and physical-mixture catalysts exhibit enhanced activity. Reaction conditions: 0.1 M NaOH; 0.02 M HMF; 50 ml H_2_O; 25 °C; scan rate: 50 mV s^−1^; N_2_ flow: 50 ml min^−1^. **b** Thermochemical performance of the same Au and Pd catalyst series for HMF oxidation. Reaction conditions: 0.1 M HMF; 0.4 M NaHCO_3_; 16 ml H_2_O; 80 °C; *p*O_2_ = 3 bar; 15 min; the catalyst amounts for the Au/C + Pd/C physical mixture, Au/Pd/C spatial separation and Au-Pd/C alloy were 143 mg; 72 mg of Au/C and 71 mg of Pd/C. For each reaction, the molar ratio of Au to Pd was maintained at 4:1; reaction time: 15 min. **c** Linear correlation between the thermochemical and electrochemical catalytic activities. HMF: 5-hydroxymethylfurfural; FDCA: 2,5-furandicarboxylic acid; FFCA: formyl-2-furancarboxylic acid; HMFCA: 5-hydroxymethyl-2-furancarboxylic acid. The associated error bars correspond to mean ± s.d. (*n*  =  3). Reproduced with permission from ref. ^18^. Copyright 2022 Springer Nature. The associated error bars correspond to mean ± s.d. (*n*  =  3).

Remarkably, this enhancement, reflected by both the increased current density measured by cyclic voltammetry and the higher product yield obtained from the thermochemical process, follows a consistent catalytic trend: Au/Pd spatial separation >Au + Pd physical mixture >Au-Pd alloy > Au > Pd (Fig. 4a, b). In this case, the low activity of Pd can be attributed to several factors, including (1) a low metal loading: in our previous work, the Pd loading was only 0.25 wt%^18^, whereas a higher loading of typically 1 wt% is commonly used in the literature; (2) The Pd nanoparticles were synthesized in the presence of a capping agent (poly(vinyl alcohol)(PVA) or poly(vinylpyrrolidone)(PVP); Pd:capping agent weight ratio = 1:1), which may remain on the surface and contribute to the deactivation of HMF oxidation.; (3) Reaction time: The reaction was carried out for only 15 min in order to determine the initial reaction rate. Moreover, the electro- and thermocatalytic results exhibit an approximately linear correlation, as shown in Fig. 4c. Such correlation is not unexpected, as electrochemical behavior is widely recognized as a useful predictor of thermocatalytic performance. The enhanced performance over the physical mixture catalysts is attributed to the efficient transfer of electrons through either static interparticle contact or frequent collisions at the physical interfaces between distinct Au/C and Pd/C catalyst particles, without perturbing the intrinsic electrochemical potentials of the metals. Specifically, the oxidation of alcohol substrates occurs on the Au surface under alkaline conditions, where the generated electrons are subsequently transferred to adjacent Pd nanoparticles to drive the four-electron oxygen reduction reaction (ORR). The resulting OH^−^ species replenishes the consumed hydroxide ions in the alkaline electrolyte, thereby closing the catalytic cycle. The oxidation of HMFCA and FFCA occurs in the same way on the Au surface. Based on this mechanism, we propose that interparticle collisions enhance the effective proximity between Au and Pd nanoparticles particularly in the physical mixture, facilitating rapid shuttling of reactive electrons and ultimately governing the overall rate of the HMF oxidation.

The promoted cooperation between two metals becomes more pronounced for the spatially separated catalyst, in which the catalyst architecture with Au and Pd nanoparticles co-supported on a common support facilitate the electron transfer. In this configuration, the transfer of electrons no longer relies primarily on stochastic interparticle collisions, and the reaction does not occur exclusively at transient physical interfaces between distinct catalytic counterparts. These observations suggest that electron transfer limits the reaction in each elementary step. Accordingly, the rational design of physical-mixture or spatially separated catalysts facilitates this transfer process and lowers the overall reaction energy barrier.

### Metal Ostwald ripening compromises the stability of spatially segregated catalysts

**Fig. 5:**
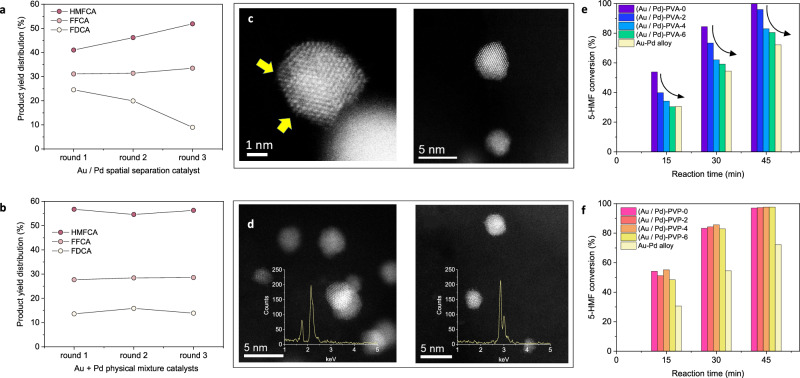
Structural stability and catalytic durability of spatially separated versus physically mixed catalyst during thermochemical 5-hydroxymethylfurfural oxidation. Catalytic stability tests over multiple reaction cycles for (**a**) the spatially separated catalyst and (**b**) the physically mixed catalysts. Reaction conditions: 0.1 M HMF; 0.4 M NaHCO_3_; 16 ml H_2_O; 80 °C; *p*O_2_ = 3 bar; 60 min; the catalyst amounts for the Au/C + Pd/C physical mixture were 72 mg of Au/C and 71 mg of Pd/C. For the Au/Pd/C spatially separated catalyst, the total amount used was 143 mg. Corresponding HAADF-STEM images of (**c**) the spatially separated catalyst and (**d**) the physically mixed catalyst. Au and Pd nanoparticles remain spatially separated after one reaction cycle in the physically mixed catalyst, insets in **d** are the XEDS spectra of the corresponding particle, showing the presence of peaks of Au M (approximately 2.1 keV) and Pd L (approximately 2.8 keV). In contrast, yellow arrows in (c) highlight atomic columns with reduced contrast, indicating partial alloying of Pd with the Au matrix after one reaction cycle in the spatially separated catalyst. Spatially separated Au and Pd nanoparticles aged for *X* hours (*X* = 0, 2, 4, and 6) with PVA (**e**) and PVP (**f**) before immobilizing on the carbon support and their catalytic performance in thermochemical HMF oxidation, compared with that of the Au-Pd alloy catalyst. Same reaction conditions to (**a**, **b**). HMFCA hydroxymethyl-2-furancarboxylic acid, FFCA formyl-2-furancarboxylic acid, FDCA furandicarboxylic acid, PVA PVP; (**a**, **b**, **c**, **f**) Reproduced with permission from ref. ^18^. Copyright 2022 Springer Nature. **d**, **e** Reproduced from the author’s PhD thesis, ref. ^30^. © Xiaoyang Huang, 2020.

Despite its enhanced initial activity, spatially separated and co-supported catalysts are inherently limited by a thermodynamic drive towards alloying. Under reaction conditions, particularly in thermocatalytic processes involving elevated temperatures, metal nanoparticles fixed on a common support eventually migrate and undergo Ostwald ripening^48–50^. The consequences of this structural evolution are clearly demonstrated in Fig. 5a, where the spatially separated Au/Pd catalyst exhibits a marked decline in activity during the second and third reaction cycles and produce lower yields of the final product FDCA. This indicates that some in situ-formed Au-Pd alloy nanoparticles featuring a Janus-like structure within spatially separated Au/Pd catalyst are more selective for HMFCA production. It has been widely reported that during the oxidation of HMF, the subsequent oxidation of the intermediate HMFCA to FFCA is the rate-determining step^57^. Therefore, the Au-Pd alloy catalyst shows lower activation of this step, resulting in the formation of HMFCA as the major product. In contrast, the physically mixed catalyst (Fig. 5b) displays substantially greater stability, maintaining nearly identical product distributions over three consecutive cycles. This implies that the Au and Pd nanoparticles remain spatially separated. We attribute this difference in performance to the distinct structural evolution of each system.

In case of the spatially separated catalyst, high-resolution STEM reveals coalescence of the segregated metals after the reaction into less active bimetallic structures, such as Janus or core-shell particles (Fig. 5c). Conversely, the physically mixed structure introduces a macroscopic geometric barrier which is caused by segregation of Au and Pd populations onto distinct support grains. As confirmed in Fig. 5d, the metal nanoparticles in the physically mixed catalyst remain spatially isolated even after repeated cycling, effectively eliminating the diffusion pathway which is required for Ostwald ripening.

To further validate the assumption that preventing metal-metal contact is the key to improved stability, we previously investigated the role of PVA and PVP as surface ligands and its effect on the structural evolution during aging^18,30^. As shown in Fig. 5e, PVA-protected colloids, which offer weaker steric hindrance, permit strong metal-metal interactions, leading to rapid alloying^53^ and a progressive decline in HMF conversion as aging time increases. However, when a robust molecular barrier is introduced using PVP, re-alloying is efficiently suppressed. Consequently, PVP-protected colloids maintain consistently high HMF conversion rates, independent of the aging time (Fig. 5f). These results confirm that maintaining a rigid barrier against alloying, either by using molecular ligands such as PVP or macroscopic segregation in form of physical mixing, is the fundamental prerequisite for stable cooperative catalysis.

### Electronic connectivity between metals is the cooperative redox enhancement mechanism

Enhancing metal-metal interactions is one of the most important strategies in catalyst design. In the previous two sections, we introduced the fundamental concepts, catalytic performance, and limitations of spatially separated catalysts and physical mixture catalysts. Although these two catalytic systems differ from metal alloy catalysts, where multiple metal atoms are assembled at the atomic level within a single nanoparticle, the interactions between metal nanoparticles remain a central consideration in the design of dealloyed catalysts.

**Fig. 6:**
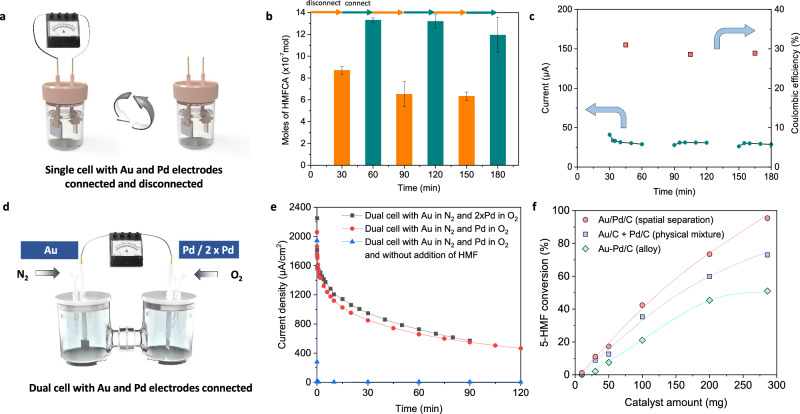
Mechanistic validation of cooperative redox enhancement via macroscale electrical connectivity between two metals in physically mixed catalysts. **a** Schematic illustration of application of Au/C and Pd/C catalyst as two electrodes for thermochemical HMF oxidation in a single cell with periodic electrical connection and disconnection via an amperemeter. Corresponding temporal evolution of (**b**) the number of HMFCA generated and (**c**) the current measured. The associated error bars in (**b**) correspond to mean ± s.d. (*n*  =  3). **d** Schematic illustration of application of Au/C and Pd/C catalyst for thermochemical HMF oxidation in dual cell with constant electrical connection via an amperemeter. **e** Corresponding temporal evolution of the current density recorded. **f** Evaluation of metal-metal interactions by varying the catalyst amount using a series of Au and Pd catalysts in thermocatalytic HMF oxidation. Reaction conditions are same to Fig. 5a, b. (**a**–**c**, **f**). HMFCA hydroxymethyl-2-furancarboxylic acid, HMF 5-hydroxymethylfurfural; Reproduced from the author’s PhD thesis, ref. ^30^. © Xiaoyang Huang, 2020. (**d**, **e**) Reproduced with permission from ref. ^18^. Copyright 2022 Springer Nature.

For spatially separated catalysts, metal active sites are statistically anchored on the support. From a dynamic perspective, physically mixed catalysts therefore exhibit unique advantages, as metal-metal interactions can occur through collision processes in which two or more metal nanoparticles transiently come into contact. As shown in Fig. 6a–c, Au/C and Pd/C were employed as the working and counter electrodes, respectively, for thermocatalytic HMF oxidation. The two electrodes were periodically disconnected and reconnected using an amperemeter, with each state maintaining for 30 min (Fig. 6a). Notably, when the two catalysts were electrically connected, the yield of the conversion of HMF into the first reaction intermediate HMFCA was approximately two times higher than when the electrodes were disconnected (Fig. 6b). Meanwhile, based on the current recorded by the amperemeter, the electron transfer efficiency between Au and Pd was calculated to be approximately 30% (Fig. 6c)^30^. Here, the electron transfer efficiency (i.e., charge transfer) for single and dual cells is defined as the ratio of the measured electron quantity flowing from the Au/C electrode to the Pd/C electrode and the theoretical electron quantity which should be generated based on the reaction stoichiometry determined by high performance liquid chromatogaphy (HPLC) analysis of the products in the Au/C electrode compartment after the reaction:

$${Electron}\,{transfer}\,{efficiency}(\%)=({{\rm{the}}}\,{{\rm{measured}}}\,{{\rm{electron}}}\,{{\rm{quantity}}}/ \\ {{\rm{the}}}\,{{\rm{stoichiometric}}}\; {{\rm{electron}}}\; {{\rm{quantity}}})*100$$

The quantity of electrons, i.e., moles of electrons, transferred between two electrodes via the amperemeter was obtained using the equation below, where *Q* (*C*) is the amount of charge (A × s) measured during the time (s) of the reaction and F the Faraday constant (96,485 C/mol):

1 $${{\rm{n}}}({{\rm{electrons}}})_{{\rm{transfer}}}={{\rm{Q}}}/{{\rm{F}}}$$

*Q* is obtained by integrating the curve (current vs. time) recorded during the reaction. The stoichiometric quantity of electrons generated during the reaction is twice the quantity of HMFCA detected by HPLC:

2 $${{\rm{n}}}\,({{\rm{electrons}}})\,_{{\rm{stoichiometric}}}=2 * {{\rm{n}}}\,({\mathrm{HMFCA}})$$

This result suggests that approximately 30% of the electrons released during HMF oxidation on the Au surface were transferred to Pd sites to drive the ORR, indicating the presence of cooperative behavior between the physically mixed catalysts. The remaining 70% loss in Au-Pd coupling efficiency can be attributed to oxygen scavenging on the Au surface as well as resistive loss within the electrochemical cell. To further strengthen metal-metal interactions and thereby to increase the electron transfer efficiency, Au/C and Pd/C electrodes were deposited separately in a dual-cell configuration and connected via an amperemeter. Nitrogen and oxygen were supplied to the Au and Pd cells, respectively (Fig. 6d). Under these conditions, the electron transfer efficiency increased to 65%, and further rose to 82% upon doubling the Pd loading (Fig. 6e). No current was detected in the absence of HMF, confirming that the observed response originates from HMF oxidation^18^. We propose that, over relatively short reaction times, near-100% efficiency is achievable, meaning that all electrons generated by the oxidation reaction at one electrode can be released and collected by the adjacent electrode to drive the corresponding reduction reaction within the electrochemical cell. However, metal leaching into the electrolyte is unavoidable. Although strong metal-support interactions can be engineered to significantly slow the leaching process, prolonged reaction times will inevitably result in a decrease in metal content, analogous to Ostwald ripening, thereby weakening metal-metal interactions. Collectively, these electrochemical experiments elucidate the role of each metal and the nature of the CORE effect in physically mixed catalysts, highlighting that electrical connectivity through the support is critical. The dynamic nature of DICC is further evidenced by the dependence of catalytic performance on mechanical agitation. As shown in Fig. 6f, both the spatially separated catalyst and the physical mixture catalyst exhibit a linear correlation between catalyst mass and HMF conversion, suggesting that the reactions proceed without significant mass transfer limitations. In contrast, the activity of the alloy catalyst quickly levels off with increasing catalyst dose, indicating the presence of diffusion limitations^30^. This observation provides an important insight into the catalytic behavior. For the spatially separated catalyst, the co-supported Au and Pd nanoparticles on the carbon support facilitate electron transfer between Au and Pd. For the physical mixture catalyst, the high-frequency collisions between Au/C and Pd/C particles may enable electron shuttling during each collision event, allowing the reaction to remain in the kinetic regime. However, this mechanism does not apply to the alloy catalyst, where Au and Pd atoms are integrated within the same nanoparticle. Moreover, this phenomenon draws a striking parallel to the fundamental principles of single-entity electrochemistry established by Bard and colleagues^45,46^. Their seminal work demonstrates that individual collisions of catalytic nanoparticles (e.g., Pt) with an inert electrode (e.g., Carbon) can instantaneously “switch on” electrocatalytic amplification, generating observable current steps derived from the sudden activation of the reaction at the interface. In our physical mixture system, we effectively scale up this phenomenon: the collision-enabled electron transfer operates as a macroscopic ensemble of these stochastic events. Consequently, the reaction rate is governed by the frequency of these transient “effective collisions” between the Au and Pd micro-environments. Unlike static alloys where the active site is fixed, the active site in a physical mixture is a temporal event created only when two particles interact. This collision-dependent behavior validates the physical mixture not as a disjointed system, but as a “pseudo-homogeneous” catalyst that retains the separation benefits of a heterogeneous system. We further suggest that physically mixed catalysts can exhibit activities approaching those of spatially separated catalysts upon suitable optimization, reflecting an equivalent level of metal-metal interaction. This optimization depends on factors such as interparticle collision intensity, slurry-phase operation at high catalyst concentrations, and support physicochemical properties, including surface area and electrochemical conductivity.

## Outlook

Carbon neutrality, a central target of the United Nations Sustainable Development Goals, is intrinsically linked to the optimization of global energy systems, particularly the transition from fossil-fuel dependence to sustainable energy utilization^58,59^. In this transformation, heterogeneous catalysis has assumed an increasingly critical role. Over the past five decades, catalytic research evolved from alloy catalysts where strong metal-metal interactions underpin many industrial processes to single-site catalysts that maximize atomic efficiency. More recently, catalysts based on spatial separation or physical mixtures started to attract attention and may represent an emerging concept in catalytic science.

To move beyond incremental advances, we propose that metal-metal separation, rather than direct alloying, can drive a distinct and potentially stronger form of catalytic synergy. In this Perspective, we highlight the unique advantages of physical mixture catalysts and outline three key directions for their future development.

First, from a mechanistic viewpoint inspired by molecular catalysis, we revisit the foundational insights of John H. Sinfelt, who pioneered bimetallic catalysis by demonstrating synergistic effects beyond simple additive behavior in alloys^60^. Extending this concept, we propose that in physical mixture catalysts with an apparent effect of 1 + 1 > 2, the catalytically relevant active sites are not static atomic ensembles within a single nanoparticle, but rather dynamic interfaces formed transiently between distinct particle grains during efficient collision events. We define this mechanism as DICC, which captures the time-dependent and spatially heterogeneous nature of the catalytic synergy.

Second, from a practical standpoint, physical mixture catalysts are particularly well suited for high-concentration and solvent-free reactions. Thanks to the structural robustness and resistance of physical mixture catalysts to sintering or deactivation, they offer clear advantages over atomically dispersed systems. For these cases, intermediate transfer among supported metal nanoparticles involves not only electrons but may also include radical species, i.e., H**·** in organic-phase reactions.

Finally, the application scope of physical mixture and spatially separated catalysts may extend beyond traditional catalysis into energy storage systems, such as redox flow batteries. In these systems, multiple active species which are not limited to Au and Pd, such as many other metals or metal oxides (e.g., Pt, Rh, Cu, Fe, and Mn) can be independently anchored onto carbon felt-based electrodes, enabling precise modulation of the redox peak potential separation (△*E*), the electrochemical voltage window of the full cell and Coulombic efficiency for redox-active organic molecules.

## References

[1] Tway, C. L. & Filip, S. V. Catalysis at the crossroads. *Science* **388**, 29–30 (2025). 40179171 10.1126/science.adw5529

[2] Liu, L. & Corma, A. Metal catalysts for heterogeneous catalysis: from single atoms to nanoclusters and nanoparticles. *Chem. Rev.* **118**, 4981–5079 (2018). 29658707 10.1021/acs.chemrev.7b00776 PMC6061779

[3] Zhao, Y. et al. Solar- versus thermal-driven catalysis for energy conversion. *Joule* **3**, 920–937 (2019).

[4] Fujishima, A. & Honda, K. Electrochemical photolysis of water at a semiconductor electrode. *Nature* **238**, 37–38 (1972). 12635268 10.1038/238037a0

[5] Sajwan, D., Sharma, A., Sharma, M. & Krishnan, V. Upcycling of plastic waste using photo-, electro-, and photoelectrocatalytic approaches: a way toward circular economy. *ACS Catal.* **14**, 4865–4926 (2024).

[6] Hörold, S., Tacke, T. & Vorlop, K. Catalytical removal of nitrate and nitrite from drinking water: 1. Screening for hydrogenation catalysts and influence of reaction conditions on activity and selectivity. *Environ. Technol.* **14**, 931–939 (1993).

[7] Corma, A., Iborra, S. & Velty, A. Chemical routes for the transformation of biomass into chemicals. *Chem. Rev.* **107**, 2411–2502 (2007). 17535020 10.1021/cr050989d

[8] Oh, R. et al. Insights into CeO_2_ particle size dependent selectivity control for CO_2_ hydrogenation using Co/CeO_2_ catalysts. *ACS Catal.* **14**, 897–906 (2024).

[9] Huang, X. J. et al. Advances in physical mixture catalysts as a heterogeneous catalytic strategy: a review. *ACS Catal.* **15**, 12204–12221 (2025).

[10] Meng, F. et al. Rational design of SAPO-34 zeolite in bifunctional catalysts for syngas conversion into light olefins. *Ind. \& Eng. Chem. Res.* **61**, 11397–11406 (2022).

[11] Bell, A. T. The impact of nanoscience on heterogeneous catalysis. *Science* **299**, 1688–1691 (2003). 12637733 10.1126/science.1083671

[12] Nørskov, J. K., Bligaard, T., Rossmeisl, J. & Christensen, C. H. Towards the computational design of solid catalysts. *Nat. Chem.* **1**, 37–46 (2009). 21378799 10.1038/nchem.121

[13] Tedsree, K. et al. Hydrogen production from formic acid decomposition at room temperature using a Ag–Pd core–shell nanocatalyst. *Nat. Nanotechnol.* **6**, 302–307 (2011). 21478867 10.1038/nnano.2011.42

[14] Yan, W. et al. Ga-modification near-surface composition of Pt–Ga/C catalyst facilitates high-efficiency electrochemical ethanol oxidation through a C2 intermediate. *J. Am. Chem. Soc.* **145**, 17220–17231 (2023). 37492900 10.1021/jacs.3c04320

[15] Zhang, H. et al. Computational and experimental demonstrations of one-pot tandem catalysis for electrochemical carbon dioxide reduction to methane. *Nat. Commun.* **10**, 3340 (2019). 31350416 10.1038/s41467-019-11292-9 PMC6659690

[16] Sankar, M. et al. Designing bimetallic catalysts for a green and sustainable future. *Chem. Soc. Rev.* **41**, 8099–8139 (2012). 23093051 10.1039/c2cs35296f

[17] Toshima, N. & Yonezawa, T. Bimetallic nanoparticles—novel materials for chemical and physical applications. *New J. Chem.* **22**, 1179–1201 (1998).

[18] Huang, X. et al. Au–Pd separation enhances bimetallic catalysis of alcohol oxidation. *Nature* **603**, 271–275 (2022). 35038718 10.1038/s41586-022-04397-7

[19] Daniel, I. T. et al. Uncovering cooperative redox enhancement effects in bimetallic catalysis. *Acc. Chem. Res.* **58**, 3235–3246 (2025). 41118286 10.1021/acs.accounts.5c00446 PMC12590465

[20] Kim, B. et al. Galvanic coupling measurements are a predictive tool for cooperative redox enhancement (CORE) in thermocatalytic alcohol oxidation. *ACS Catal.* **15**, 18063–18068 (2025).

[21] Li, X. et al. Solvent-free benzyl alcohol oxidation using spatially separated carbon-supported Au and Pd nanoparticles. *ACS Catal.* **14**, 16551–16561 (2024).

[22] Lodaya, K. M. et al. An electrochemical approach for designing thermochemical bimetallic nitrate hydrogenation catalysts. *Nat. Catal.* **7**, 262–272 (2024).

[23] Liang, Y. et al. Boosting selective hydrogenation of α,β-unsaturated aldehydes through constructing independent Pt and Fe active sites on support. *Chem. Eng. J.* **484**, 149670 (2024).

[24] Ge, H. et al. Pt–Co separation for enhancing bimetallic catalysis in selective hydrogenation reaction. *ACS Catal.* **15**, 16740–16747 (2025).

[25] Zhang, P. et al. Synergy induced by physical mixing m-ZrO_2_ and α-Ga_2_O_3_ for effective conversion of syngas to light olefins. *J. Catal.* **442**, 115934 (2025).

[26] Zhang, P. et al. Tandem reactions on phase separated MnO_2_ and C to enhance formaldehyde conversion to hydrogen. *Int. J. Hydrogen Energy* **51**, 982–992 (2024).

[27] Fang, W. et al. Physical mixing of a catalyst and a hydrophobic polymer promotes CO hydrogenation through dehydration. *Science* **377**, 406–410 (2022). 35862543 10.1126/science.abo0356

[28] McIntosh, S. Revealing hidden nanoscale electrocatalysis. *Nat. Catal.* **8**, 287–288 (2025).

[29] Peng, Y. et al. To alloy or not to alloy? The unexpected power of Pd–Au catalyst physical mixtures in efficient HMF oxidation to FDCA. *ACS Catal.* **15**, 11760–11773 (2025).

[30] Huang, X. *Investigations of Interaction Between Gold and Palladium in Alcohol Oxidation Reactions* (Cardiff University, 2020).

[31] Karim, W. et al. Catalyst support effects on hydrogen spillover. *Nature* **541**, 68–71 (2017). 28054605 10.1038/nature20782

[32] Lim, K. R. G. et al. Nanoparticle proximity controls selectivity in benzaldehyde hydrogenation. *Nat. Catal.* **7**, 172–184 (2024).

[33] Kumar, S. & Zou, S. Electrooxidation of CO on uniform arrays of Au nanoparticles: effects of particle size and interparticle spacing. *Langmuir* **25**, 574–581 (2009). 19063641 10.1021/la802747g

[34] Inaba, M. et al. The oxygen reduction reaction on Pt: why particle size and interparticle distance matter. *ACS Catal.* **11**, 7144–7153 (2021).

[35] Daniel, I. T. et al. Kinetic analysis to describe Co-operative redox enhancement effects exhibited by bimetallic Au–Pd systems in aerobic oxidation. *Catal. Sci. Technol.* **13**, 47–55 (2023).

[36] Zhao, L. et al. Insights into the effect of metal ratio on cooperative redox enhancement effects over Au- and Pd-mediated alcohol oxidation. *ACS Catal.* **13**, 2892–2903 (2023). 36910870 10.1021/acscatal.2c06284 PMC9990151

[37] Daniel, I. T. et al. Electrochemical polarization of disparate catalytic sites drives thermochemical rate enhancement. *ACS Catal.* **13**, 14189–14198 (2023). 37942270 10.1021/acscatal.3c03364 PMC10631442

[38] Kim, B. et al. Tafel analysis predicts cooperative redox enhancement effects in thermocatalytic alcohol dehydrogenation. *ACS Catal.* **14**, 8488–8493 (2024).

[39] Kim, B. et al. Designing physically separated bimetallic catalysts through cooperative redox enhancement (CORE). *Chem. Soc. Rev.* **55**, 1293–1305 (2026). 41481266 10.1039/d4cs00479e

[40] Zhang, J.-J., Lou, Y.-Y., Wu, Z., Huang, X. J. & Sun, S.-G. Spatially separated Cu/Ru on ordered mesoporous carbon for superior ammonia electrosynthesis from nitrate over a wide potential window. *J. Am. Chem. Soc.* **146**, 24966–24977 (2024). 39197103 10.1021/jacs.4c06657

[41] Parlett, C. M. A. et al. Spatially orthogonal chemical functionalization of a hierarchical pore network for catalytic cascade reactions. *Nat. Mat.* **15**, 178–182 (2016). 10.1038/nmat4478 26569475

[42] Corma, A. Separate to accumulate. *Nat. Mat.* **15**, 134–136 (2016). 10.1038/nmat4537 26796730

[43] Isaacs, M. A. et al. A spatially orthogonal hierarchically porous acid–base catalyst for cascade and antagonistic reactions. *Nat. Catal.* **3**, 921–931 (2020).

[44] Hammer, B. & Nørskov, J. K. Theoretical surface science and catalysis—calculations and concepts. *Adv. Catal.* **45**, 71–129 (2000).

[45] Xiao, X. & Bard, A. J. Observing single nanoparticle collisions at an ultramicroelectrode by electrocatalytic amplification. *J. Am. Chem. Soc.* **129**, 9610–9612 (2007). 17630740 10.1021/ja072344w

[46] Bard, A. J., Zhou, H. & Kwon, S. J. Electrochemistry of single nanoparticles via electrocatalytic amplification. *Isr. J. Chem.* **50**, 267–276 (2010).

[47] Anderson, T. J. & Zhang, B. Single-nanoparticle electrochemistry through immobilization and collision. *Acc. Chem. Res.* **49**, 2625–2631 (2016). 27730817 10.1021/acs.accounts.6b00334 PMC5518676

[48] Hansen, T. W., Delariva, A. T., Challa, S. R. & Datye, A. K. Sintering of catalytic nanoparticles: particle migration or Ostwald ripening? *Acc. Chem. Res.* **46**, 1720–1730 (2013). 23634641 10.1021/ar3002427

[49] Campbell, C. T. The energetics of supported metal nanoparticles: relationships to sintering rates and catalytic activity. *Acc. Chem. Res.* **46**, 1712–1719 (2013). 23607711 10.1021/ar3003514

[50] Jennings, D. et al. Direct atomic-scale investigation of the coarsening mechanisms of exsolved catalytic Ni nanoparticles. *Nat. Commun.* **16**, 6830 (2025). 40707489 10.1038/s41467-025-61971-z PMC12290124

[51] Liu, R. & Priestley, R. D. Rational design and fabrication of core–shell nanoparticles through a one-step/pot strategy. *J. Mater. Chem. A* **4**, 6680–6692 (2016).

[52] Wang, D. & Li, Y. Bimetallic nanocrystals: liquid-phase synthesis and catalytic applications. *Adv. Mater.* **23**, 1044–1060 (2011). 21218429 10.1002/adma.201003695

[53] Munnik, P., de Jongh, P. E. & de Jong, K. P. Recent Developments in the Synthesis of Supported Catalysts. *Chem. Rev.* **115**, 6687–6718 (2015). 26088402 10.1021/cr500486u

[54] Oh, R. et al. Enhanced alcohol electrochemical oxidation by using an environmentally friendly xanthan gum binder. *ACS Sustain. Chem. Eng.* **11**, 11681–11692 (2023).

[55] Pham, T. H. M. et al. Enhanced electrocatalytic CO_2_ reduction to C2+ products by adjusting the local reaction environment with polymer binders. *Adv. Energy Mater.* **12**, 2103663 (2022).

[56] Matthews, G. et al. Impact of binder content on particle fracture and microstructure of solvent-free electrodes for Li-ion batteries. *J. Mater. Chem. A* **13**, 18283–18291 (2025).

[57] Chen, Y. et al. Oxygen vacancy-induced metal–support interactions in aupd/zro2 catalysts for boosting 5-hydroxymethylfurfural oxidation. *Inorg. Chem.* **62**, 15277–15292 (2023). 37656824 10.1021/acs.inorgchem.3c02473

[58] DPI, U. N. *The Sustainable Development Goals* (Stylus Publishing, LLC, 2017).

[59] IRENA. *World Energy Transitions Outlook 2023: 1.5 °C Pathway*(International Renewable Energy Agency, 2023).

[60] Sinfelt, J. H. Supported “bimetallic cluster” catalysts. *J. Catal.* **29**, 308–315 (1973).

